# Effects of alterations in positron emission tomography imaging parameters on radiomics features

**DOI:** 10.1371/journal.pone.0221877

**Published:** 2019-09-05

**Authors:** Rachel B. Ger, Joseph G. Meier, Raymond B. Pahlka, Skylar Gay, Raymond Mumme, Clifton D. Fuller, Heng Li, Rebecca M. Howell, Rick R. Layman, R. Jason Stafford, Shouhao Zhou, Osama Mawlawi, Laurence E. Court

**Affiliations:** 1 Department of Radiation Physics, The University of Texas MD Anderson Cancer Center, Houston, Texas, United States of America; 2 MD Anderson Cancer Center UTHealth Graduate School of Biomedical Sciences, Houston, Texas, United States of America; 3 Department of Imaging Physics, The University of Texas MD Anderson Cancer Center, Houston, Texas, United States of America; 4 Department of Radiology, Texas Children’s Hospital, Houston, Texas, United States of America; 5 Department of Radiation Oncology, The University of Texas MD Anderson Cancer Center, Houston, Texas, United States of America; 6 Department of Biostatistics, The University of Texas MD Anderson Cancer Center, Houston, Texas, United States of America; University of Central Florida (UCF), UNITED STATES

## Abstract

Radiomics studies require large patient cohorts, which often include patients imaged using different imaging protocols. We aimed to determine the impact of variability in imaging protocol parameters and interscanner variability using a phantom that produced feature values similar to those of patients. Positron emission tomography (PET) scans of a Hoffman brain phantom were acquired on GE Discovery 710, Siemens mCT, and Philips Vereos scanners. A standard-protocol scan was acquired on each machine, and then each parameter that could be changed was altered individually. The phantom was contoured with 10 regions of interest (ROIs). Values for 45 features with 2 different preprocessing techniques were extracted for each image. To determine the impact of each parameter on the reliability of each radiomics feature, the intraclass correlation coefficient (ICC) was calculated with the ROIs as the subjects and the parameter values as the raters. For interscanner comparisons, we compared the standard deviation of each radiomics feature value from the standard-protocol images to the standard deviation of the same radiomics feature from PET scans of 224 patients with non-small cell lung cancer. When the pixel size was resampled prior to feature extraction, all features had good reliability (ICC > 0.75) for the field of view and matrix size. The time per bed position had excellent reliability (ICC > 0.9) on all features. When the filter cutoff was restricted to values below 6 mm, all features had good reliability. Similarly, when subsets and iterations were restricted to reasonable values used in clinics, almost all features had good reliability. The average ratio of the standard deviation of features on the phantom scans to that of the NSCLC patient scans was 0.73 using fixed-bin-width preprocessing and 0.92 using 64-level preprocessing. Most radiomics feature values had at least good reliability when imaging protocol parameters were within clinically used ranges. However, interscanner variability was about equal to interpatient variability; therefore, caution must be used when combining patients scanned on equipment from different vendors in radiomics data sets.

## Introduction

Radiomics involves evaluating images on a voxel level to extract quantitative image features (i.e. texture). This process relies on the assumption that there is more information contained within the images than the human eye can extract and these textures and patterns are related to the gene microenvironment within that tumor or tissue.[1] Interest in radiomics has grown as radiomics features have been shown to improve cancer survival models when combined with conventional prognostic factors (e.g., age).[2–9]

While most radiomics studies originally focused on computed tomography (CT) images, radiomics features from positron emission tomography (PET) images have also been correlated with patient survival. For example, Fried et al. [3, 10] identified radiomics features that were correlated with survival in patients with lung cancer, and they were able to identify features that distinguished subgroups of patients received a clinical benefit from radiotherapy dose escalation.

However, variability in imaging protocols can add noise to radiomics data in patient studies. For PET images, acquisition and reconstruction parameters have been shown to affect radiomics features. In particular, the number of iterations, matrix size, and smoothing filter produce variability in radiomics features.[11–21] In general, these studies have been performed using only one scanner and have investigated only a few of the parameters that can be altered in the imaging protocol. Those studies that used a phantom often used one with uniform spheres, such as the National Electrical Manufacturers Association phantom, which may not be representative of the texture within patients’ tumors. Thus, although these studies have provided valuable insight into particular issues, they may not be generalizable.

In this study, we aimed to fill this gap by using a phantom that provided radiomics feature values similar to those found in patients. We used scanners from several different vendors and investigated the effects of changing all of the parameters that could be changed for reconstructions. Filling this gap allows for more precise inclusion criteria in patient studies in order to reduce the noise in radiomics features which may produce the best possible prediction studies.

## Methods

### Phantom scans

PET images of a 3-dimensional Hoffman brain phantom were acquired on GE Discovery 710 (GE Healthcare, Chicago, IL), Siemens mCT (Siemens Healthineers, Forchheim, Germany), and Philips Vereos (Philips Healthcare, Eindhoven, The Netherlands) PET scanners. A standard-protocol scan was acquired on each machine, and then each parameter that could be changed was altered individually. For example, to assess the impact of time per bed position, the other standard-protocol parameters were held constant while the time per bed position was set to 2 minutes for one reconstruction, 3 minutes for another reconstruction, 4 minutes for another reconstruction, and 5 minutes for another reconstruction. The parameters that could be changed and the settings investigated for each scanner are listed in Table 1.

**Table 1:** Parameters changed to investigate impact on radiomics features.

	Scanner		
Parameters	GE Discovery 710	Siemens mCT	Philips Vereos
Field of view (cm)	25, 50, 70		
Filter cutoff (mm)	1, 3, 5, 8, 10	1, 3, 5, 8, 10	None, 1, 3, 5, 8, 10
Iterations × subsets	1 × 4, 2 × 8, 4 × 8, 2 × 18, 4 × 32	Non-TOF: 1 × 4, 2 × 8, 4 × 8, 2 × 12, 4 × 24 TOF: 1 × 21, 2 × 21, 3 × 21, 4 × 21	1 × 4, 2 × 8, 4 × 8, 2 × 20, 3 × 15, 4 × 32
Matrix size	128, 192, 256	128, 200, 256, 400, 512	
Time per bed position (min)	2, 3, 4, 5	2, 3, 4, 5	2, 3, 4, 5
Type of reconstruction	VPFX, VPFX-S, VPHD, VPHD-S, QCFX-S, QCHD-S	Backprojection, backprojection TOF, iterative, iterative TOF, TRUEX, TRUEX TOF	
Z smoothing	None, light, standard, heavy		

TOF: time of flight

Types of reconstruction are proprietary names used by each vendor.

The standard-protocol settings for the GE scanner were 70 cm field of view, 5 mm filter cutoff, 2 iterations and 18 subsets, 192 matrix size, standard z smoothing, 6 minutes per bed position, and VPFX-S reconstruction. The standard-protocol settings for the Siemens scanner were 82 cm field of view, 5 mm filter cutoff, 2 iterations and 21 subsets, 200 matrix size, 5 minutes per bed position, and TRUEX time-of-flight (TOF) reconstruction. As the Siemens scanner also allows for continuous bed motion, this type of acquisition was also explored and treated as an additional scanner. The standard-protocol settings were the same as the fixed number of bed positions acquisition but with 0.4 mm/s as the bed speed. The standard-protocol settings for the Philips scanner were 60 cm field of view, 3 iterations and 15 subsets, 128 matrix size, no smoothing filter, and 5 minutes per bed position. The term “standard protocol” here means that it was the baseline acquisition. The time per bed position was longer than that used clinically and for the Siemens scanner, the standard acquisition used at MD Anderson is continuous bed motion. The phantom was injected with 2.53–2.75 mCi of F-18 fluorodeoxyglucose for each scan and then imaged about 30 minutes later. The weight was set to 20 kg to obtain standardized uptake values (SUVs) in the phantom that were similar to the SUVs in patient tumors.

### Patients

Data from a patient cohort were used to provide context to the variability observed between scanners. For example, an interscanner variation of 0.4 for a given feature with a phantom does not necessarily represent the impact of interscanner variation in a patient study. However, if the interscanner variation is computed relative to interpatient variation, the impact on patient studies can be directly observed. In order to make interscanner comparisons that were relative to interpatient variation in this study, PET studies of 224 patients with non-small cell lung cancer (NSCLC) were retrospectively analyzed. The requirement for informed consent was waived and approval for use of this data was given by the Institutional Review Board at The University of Texas MD Anderson Cancer Center. This cohort consisted of 84 women and 140 men with an average age of 65 years (range, 39–89 years), average height of 171 cm (range, 147–195 cm), average weight of 82 kg (range, 39–151 kg), and average tumor volume of 90 cm^3^ (range, 0.4–920 cm^3^).

### Feature extraction

**Fig 1:**
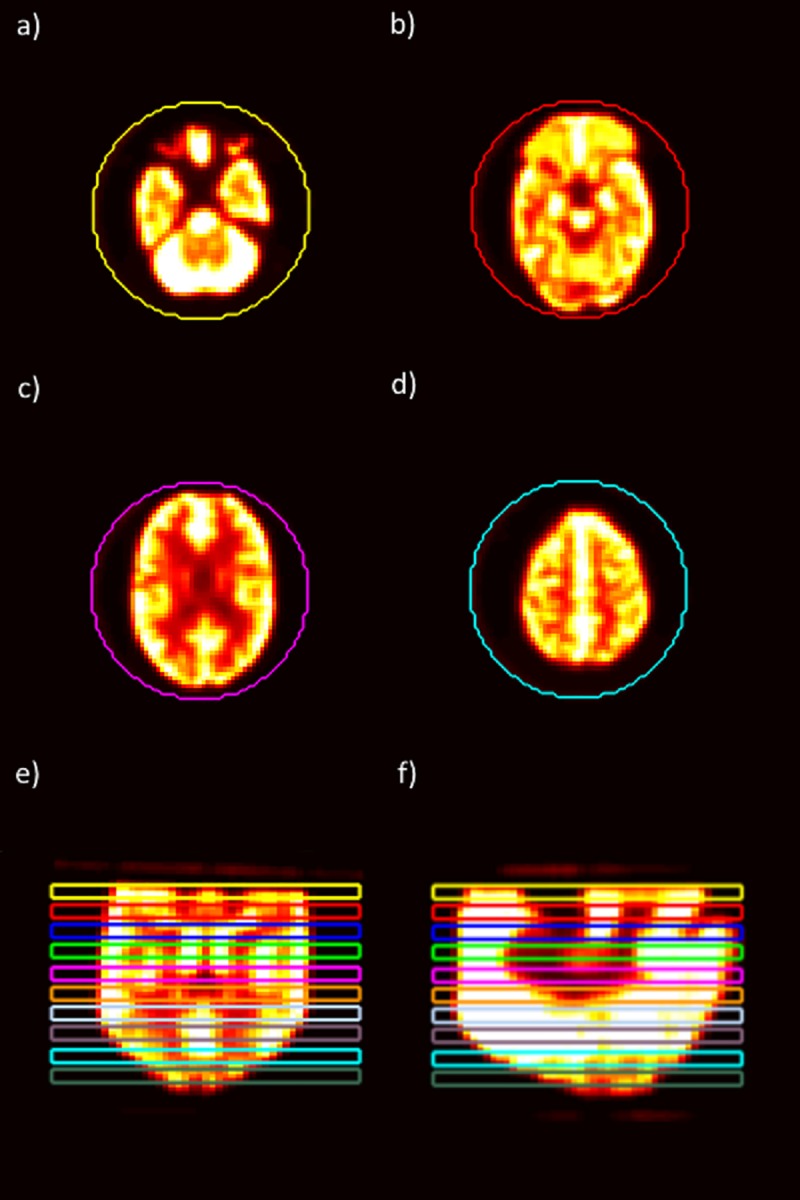
Slices of Hoffman phantom. Four slices of the Hoffman phantom are shown. Each slice is from a different ROI among the 10 ROIs that were drawn in the phantom. The example slices shown here are from different regions within the phantom: (a) near the bottom of the phantom, (b) between the bottom and the middle of the phantom, (c) between the middle and the top of the phantom, and (d) near the top of the phantom. Additionally, there are (e) coronal and (f) sagittal slices to show the placement of ROIs within the phantom.

Each phantom scan was semiautomatically contoured with 10 cylindrical regions of interest (ROIs) using in-house developed MATLAB (MathWorks, Natick, MA) scripts. Each ROI had a diameter of 19.4 cm and a height of 1 cm. Some slices of the phantom with contours are shown in Fig 1. A threshold of 0.4 SUV was used before feature calculation on the phantom images to remove background noise or activity that had leaked to the edges of the phantom container. The patient images were contoured using PET Edge in MIM (MIM Software Inc., Cleveland, OH). Forty-five features were extracted using 2 preprocessing methods: (1) a fixed-bin-width of 0.5 SUV, as suggested by Leijenaar et al.,[22] and (2) rescaling to 64 levels, as suggested by Hatt et al.[23] Both of these preprocessing methods were used as they extensively evaluated robustness of features on PET SUV images and have been used in many PET patient studies. Radiomics features were calculated using IBEX, a freely available radiomics tool.[24, 25] The features used are listed in Table 2. More information about these features can be found in a publication by Zhang et al.[24] The settings for each of the features were the same as those listed in Fave et al.’s Supplemental Material [2], except for neighborhood gray tone difference matrix, where we set the neighborhood to 3 owing to the large voxel size in PET images.

**Table 2:** Radiomics features used in PET analysis.

Gray Level Co-occurrence Matrix	Gray Level Run Length Matrix	Intensity Histogram	Neighborhood Gray Tone Difference Matrix
Auto Correlation	Gray Level Nonuniformity	Energy	Busyness
Cluster Prominence	High Gray Level Run Emphasis	Entropy	Coarseness
Cluster Shade	Long Run Emphasis	Kurtosis	Complexity
Cluster Tendency	Long Run High Gray Level Emphasis	Skewness	Contrast
Contrast	Long Run Low Gray Level Emphasis	Standard Deviation	Texture Strength
Correlation	Low Gray Level Run Emphasis	Uniformity	
Difference Entropy	Run Length Nonuniformity	Variance	
Dissimilarity	Run Percentage		
Energy	Short Run Emphasis		
Entropy	Short Run High Gray Level Emphasis		
Homogeneity	Short Run Low Gray Level Emphasis		
Homogeneity 2			
Information Measure Correlation 1			
Information Measure Correlation 2			
Inverse Difference Moment Norm			
Inverse Difference Norm			
Inverse Variance			
Max Probability			
Sum Average			
Sum Entropy			
Sum Variance			
Variance			

### Statistical analysis

The intraclass correlation coefficient (ICC) was used to determine whether changes in each parameter affected the measured radiomics features. This was done separately for each adjustable parameter on each scanner, with the ROIs as the subjects and the different parameter values as the raters. The 2-way random effects, consistency, single rater/measurement ICC as described by Shrout and Fleiss[26] was computed in R (version 3.4.3) using the psych package (version 1.7.8).[27] To determine the level of reliability indicated by the ICC values, the guidelines published by Koo and Li[28] were followed: ICC values lower than 0.5 signified poor reliability, those between 0.5 and 0.75 signified moderate reliability, those between 0.75 and 0.9 signified good reliability, and those greater than 0.9 signified excellent reliability.

Interscanner analysis was performed using the standard-protocol image from each scanner. The standard deviation across the ROIs from the four scanners (GE, Philips, Siemens, and Siemens using continuous bed motion) was compared to the standard deviation from the NSCLC patient cohort. This was done separately for each feature and preprocessing technique. Additionally, the mean value for each feature and preprocessing technique combination from the phantom standard-protocol images was compared to the mean value from the patient images for the same combination. If the phantom mean was not within two standard deviations of the patient mean for a given feature, the feature was not included when calculating the interscanner variation metric.

## Results

**Fig 2:**
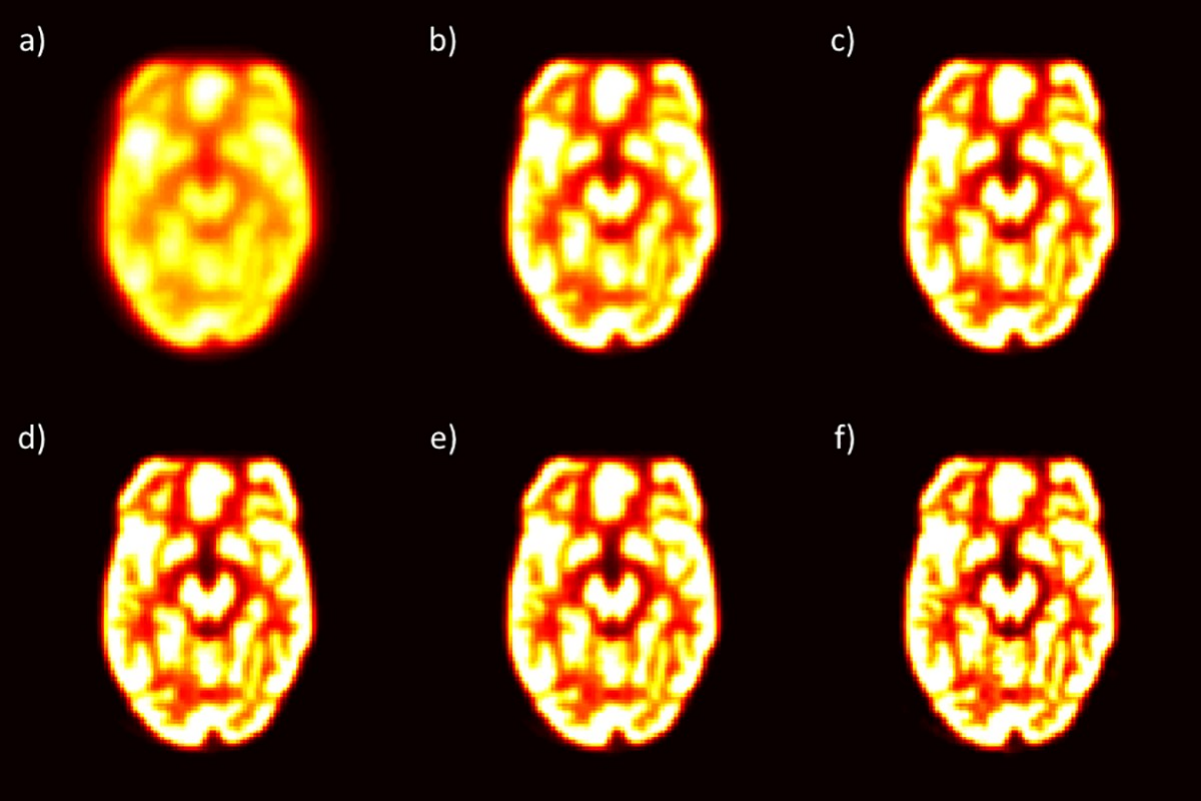
Effect of effective iterations on phantom images. One slice from the different effective iteration values (iterations × subsets) from the Philips scanner is used to demonstrate the impact of having (a) effective iterations of 4 (1 x 4), (b) effective iterations of 16 (2 x 8), (c) effective iterations of 32 (4 x 8), (d) effective iterations of 40 (2 x 20), (e) effective iterations of 45 (3 x 15), and (f) effective iterations of 128 (4 x 32).

For all scanners, most features had good (ICC > 0.75) to excellent (ICC > 0.9) reliability when reasonable parameter choices were used. Here, “reasonable” refers to parameter values that are used in clinics. For example, extremely low or extremely high effective iteration values (iterations × subsets) were excluded, as these are not actually used in clinics. An example of an extremely low effective iteration value is 4 (1 x 4), and an example of an extremely high effective iteration value is 128 (4 x 32). The low effective iteration values are not used clinically as they do not produce clear images and the high effective iteration values are not clinically used currently due to computation time. Differences in effective iteration is demonstrated in Fig 2. The following paragraphs summarize the results obtained using the reasonable parameters and give the percentage of features in each of the reliability classifications described in the Statistical Analysis section. The specific ICC values for each feature using all parameter values and the subset of parameter values that were deemed reasonable are presented in the Supplemental Material.

For the GE scanner, when the pixel size was resampled, all features had excellent reliability with both preprocessing types for field of view and matrix size. When only filter cutoff values below 6 mm were included, 96% of features had excellent reliability, 3% of features had good reliability, and 1% of features had moderate reliability (busyness calculated using fixed-bin-width preprocessing). For iterations and subsets, when only effective iterations between 16 and 36 were included, 87% of features had excellent reliability, 12% of features had good reliability, and 1% of features had poor reliability (complexity calculated using 64-level preprocessing). When time per bed position was altered, all features had excellent reliability with both preprocessing types. For the type of reconstruction, when Q.Clear was not included (reconstruction types QCFX-S and QCHD-S), 92% of features had excellent reliability and 8% of features had good reliability. For z smoothing, 89% and 11% of features had excellent and good reliability, respectively.

For the Siemens scanner, when only filter cutoff values below 6 mm were included, 80% of features had excellent reliability, 19% of features had good reliability, and 1% of features had moderate reliability (busyness calculated using fixed-bin-width preprocessing). For matrix size, when the pixel size was resampled, 94% and 6% of features had excellent and good reliability, respectively. For iterations and subsets using TOF, 83% of features had excellent reliability, 12% of features had good reliability, and 4% of features had moderate reliability. For iterations and subsets using non-TOF, when only effective iterations between 16 and 24 were included, 76% of features had excellent reliability, 18% of features had good reliability, and 7% of features had moderate reliability. For the time per bed position, all features had excellent reliability with both preprocessing types. Similar results were found using continuous bed motion.

**Fig 3:**
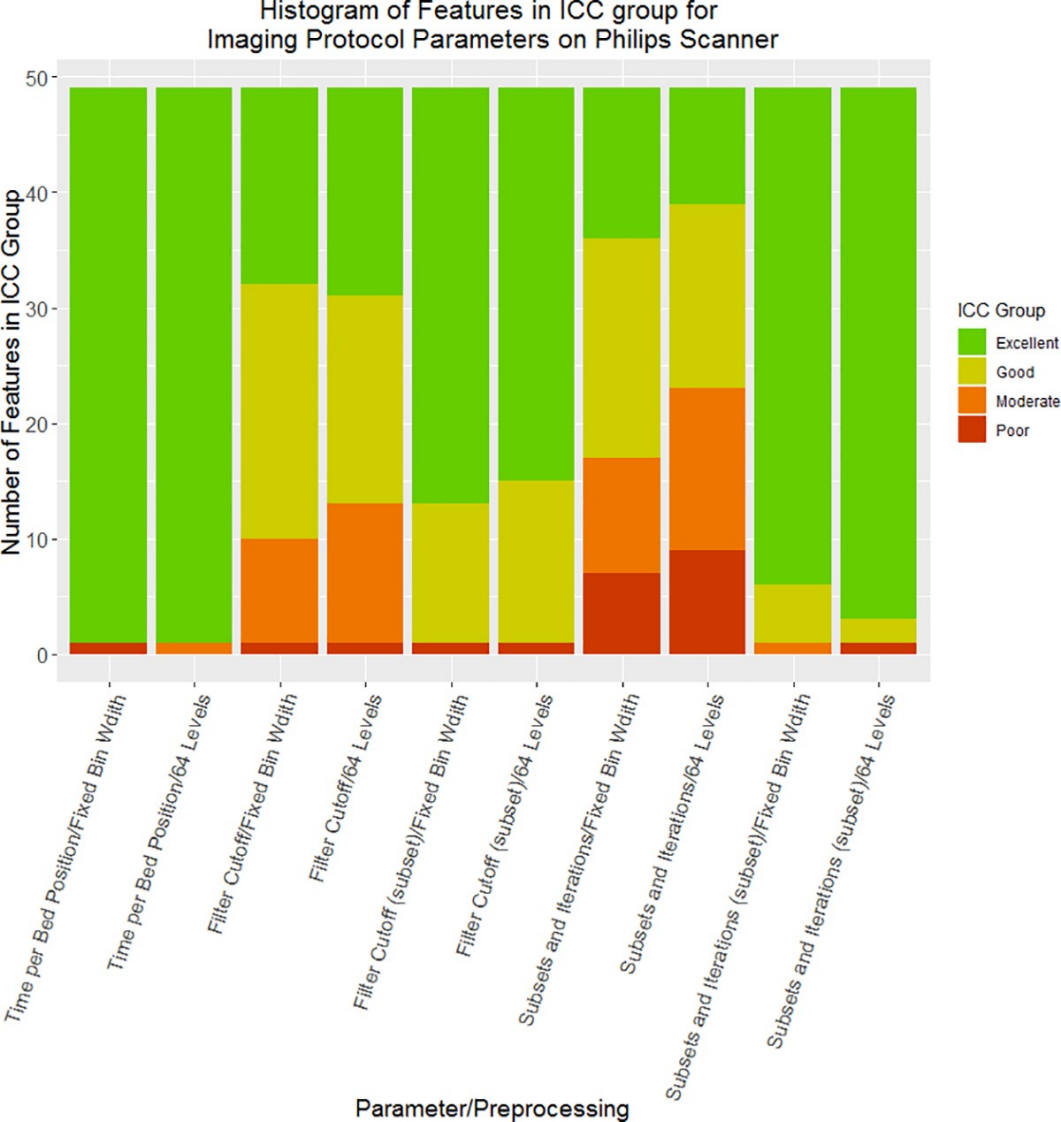
Bar plots of features by reliability level for the Philips scanner. For each imaging-protocol parameter using each of the 2 preprocessing techniques (fixed-bin-width and 64 levels), the number of features in each ICC reliability level is shown: excellent reliability (green) is ICC > 0.9, good reliability (yellow) is 0.75 < ICC < 0.9, moderate reliability (orange) is 0.5 < ICC < 0.75, and poor reliability (red) is ICC < 0.5. When parameters were limited to values seen in clinics, most features had excellent reliability, regardless of preprocessing technique. The subset for filter cutoff contains reconstructions for which the filter cutoff was below 6 mm. The subset for iterations and subsets contains reconstructions for which the effective number of iterations was between 16 and 45.

For the Philips scanner, when only filter cutoff values below 6 mm were included, 71% and 29% of features had excellent and good reliability, respectively. For iterations and subsets, when only effective iterations between 16 and 45 were included, 92% and 8% of features had excellent and good reliability, respectively. For the time per bed position, all features had excellent reliability with both preprocessing types. The distribution of features in each reliability grouping for each imaging protocol parameter and preprocessing technique for the Philips scanner is shown in Fig 3. The data used to create this figure, as well as the data for the other scanners, are detailed in the Supplemental Material.

Across all scanners, the average ICC was typically higher with fixed-bin preprocessing than with 64-level preprocessing. This was the case for all of the imaging parameters on the Siemens (both for the step-and-shoot and the continuous-bed-motion acquisition) and Philips scanners.

**Fig 4:**
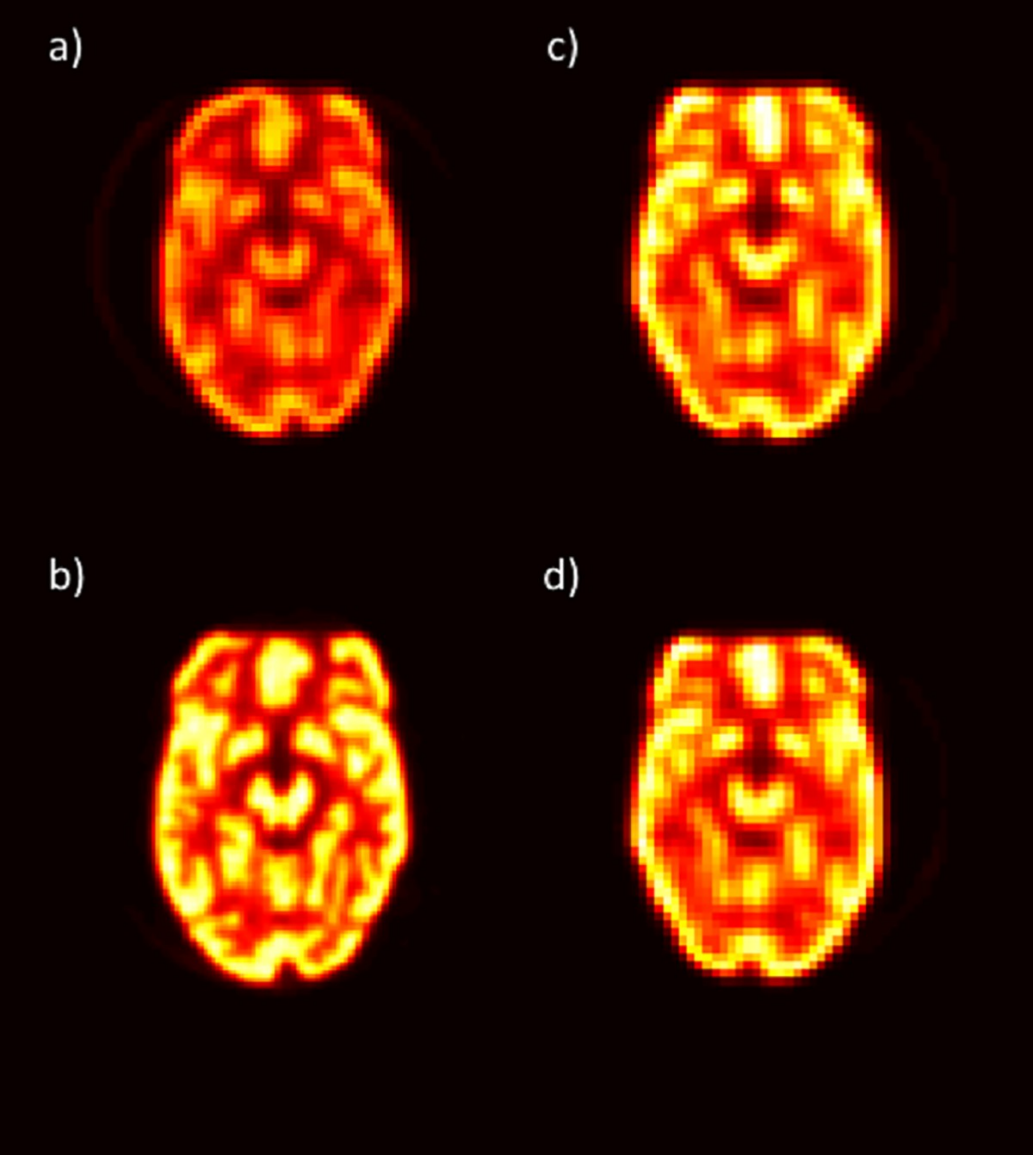
Slice from standard-protocol phantom scan. One slice from the standard-protocol phantom scan is shown for each scanner: (a) GE, (b) Philips, (c) Siemens, and (d) Siemens continuous bed motion.

One slice from the standard-protocol phantom scan used from each scanner in the interscanner analysis is shown in Fig 4. The average ratio of the standard deviation across all features from the standard-protocol phantom scans to the standard deviation from the NSCLC patient scans was 0.73 using fixed-bin-width preprocessing and 1.0 using 64-level preprocessing. With 64-level preprocessing, 7 features on the phantom scans had a mean value more than 2 standard deviations from the patient-scan mean value for that feature. Excluding these features, the mean ratio of the phantom-scan standard deviation to the patient-scan standard deviation was reduced to 0.92.

## Discussion

In this study, we investigated the impact on the variability of radiomics feature values of the imaging protocol parameters that could be retrospectively changed on GE, Philips, and Siemens PET scanners. We found that as long as reasonable parameter values were used (i.e., parameter values that are actually used in clinics), almost all features had at least good reliability. These results demonstrate that on a given scanner, radiomics data for patients scanned using different imaging protocols can be combined without adding significant noise.

However, interscanner variability was about equal to interpatient variability. This implies that caution should be used when combining data for patients scanned on equipment from different vendors for radiomics analysis as the observed stratification in a patient cohort may be due to differences in scanners and not true patient differences. A phantom with radiomics feature values similar to those of patients, such as the one used here, should be used to verify which features can be used in the patient analysis. If a phantom cannot be acquired, our full interscanner analysis, which is provided in the Supplemental Material, can serve as a reference on which features are robust enough to be included in the analysis and which features are too variable and should be excluded.

We found that there was less variability in features when using the fixed-bin-width preprocessing method than with the 64-level preprocessing method. Leijenaar et al.[22] also found that using a fixed-bin-width was preferable in their interpatient and intrapatient comparisons of two preprocessing techniques (fixed-bin-width and fixed number of levels) in 35 lung cancer patients.

When we included a large range of parameter values, our results agreed with those of previous studies that found that the parameters of filter cutoff, matrix size, and iterations and subsets affect feature values.[11–21] We were also able to show that resampling the image in our radiomics software prior to feature extraction removed the impact of matrix size on feature values. Additionally, the impact of variations in filter cutoffs and iterations and subsets could be removed if only parameter values that are commonly used in clinics were included.

This study has several limitations. First, only one scanner from each vendor was used; therefore, the variability of different models from a given vendor could not be explored. Second, only one acquisition per scanner was used for this study. We previously found the repeatability of a particular acquisition on a scanner to be very high; thus, we do not believe acquisition-level variability affected the results of this study. Third, this study was conducted using a phantom, which allowed for consistency in subject material across scanners, but the phantom is only a representation of patient texture. For this particular study, most features from the phantom, including SUV values such as SUV_mean_ and SUV_max_, were within the range of values found from the NSCLC patient cohort, showing that the phantom features were a good representation of the features and SUV values of this patient cohort. However, the activity concentration was higher in our phantom than that seen in typical patient PET scans. This could affect the convergence rate of the reconstruction algorithms. To assess the impact of convergence rates, many scans with different activity levels would have to be acquired and the whole analysis repeated, which is outside the scope of this paper. Additionally, we did not relate different features to patient outcome. This was a phantom study that aimed to determine the robustness of features to changes in PET scanner imaging parameters and not determine which features are useful as that depends on the study goal, patient population, etc. Another limitation is that, for practical reasons, we only examined a subset of the entire parameter space. For example, the voxel size relative to the filter cutoff values may also have affected the results. For the standard-protocol scan, the voxel size was 0.36 × 0.36 × 0.33 cm on the GE scanner, 0.41 × 0.41 × 0.2 cm on the Siemens scanner, and 0.2 × 0.2 × 0.2 cm on the Philips scanner. The small values of the cutoff value investigated (particularly 1 mm) would only represent part of a voxel and, would therefore, not affect the image. Smaller voxel sizes could be affected more by these filter cutoff values and could result in lower ICC values. Finally, this study used an adult NSCLC patient cohort; different adult patient cohorts may have different interpatient variability levels and different ratios of the standard deviation of the phantom measurements to that of the patients’ measurements. Results may be different in pediatric patient cohorts where the average weight is much less than the average weight of the cohort in this study.

## Conclusions

We found that all imaging-protocol parameters had good reliability across feature values when the parameter values were within limits typically used in clinics. However, interscanner variability was about equal to interpatient variability. Therefore, caution must be used when combining patients scanned using equipment from different vendors into single radiomics data sets.

## Supporting information

S1 Dataset GE phantom data. Radiomics data from the reconstructions on the GE scanner.
(XLSX)

S2 Dataset Philips phantom data. Radiomics data from the reconstructions on the Philips scanner.
(XLSX)

S3 Dataset Siemens phantom data. Radiomics data from the reconstructions on the Siemens scanner.
(XLSX)

S4 Dataset Siemens continuous bed motion phantom data. Radiomics data from the reconstructions on the Siemens scanner using continuous bed motion acquisition.
(XLSX)
